# The Solvent-Dependent Photophysics of Diphenyloctatetraene

**DOI:** 10.1021/acs.jpcb.3c03737

**Published:** 2023-09-14

**Authors:** Daniel W. Polak, Alexandros D. P. Hannon, Guilherme A. Marczak Giorio, Olivia A. Hawkins, Thomas A. A. Oliver

**Affiliations:** School of Chemistry, Cantock’s Close, University of Bristol, Bristol, BS8 1TS, U.K.

## Abstract

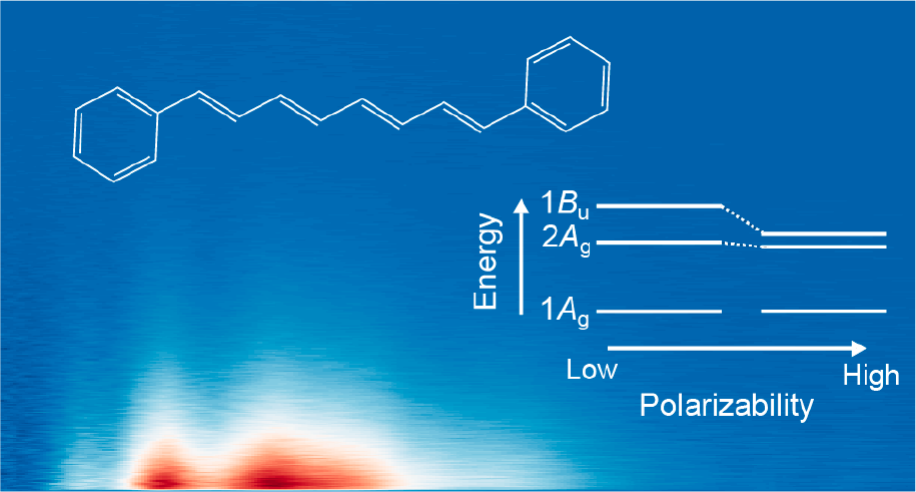

Despite many decades of study, the excited state photophysics of polyenes remains controversial. In diphenylpolyenes with conjugated backbones that contain between 2 and 4 double carbon–carbon bonds, the first two excited electronic states are nearly degenerate but of entirely different character, and their energy splitting is strongly dependent on solvent polarizability. To examine the interplay between these different states, steady-state and time-resolved fluorescence spectroscopies were used to undertake a comprehensive investigation of diphenylocatetraene’s (DPO) excited state dynamics in 10 solvents of different polarizabilities and polarities, ranging from weakly interacting alkanes to polar hydrogen-bonding alcohols. These data revealed that photopreparation of the optically bright 1*B*_u_ state resulted in fast (<170 ps) internal conversion to the lower-lying optically dark 2*A*_g_ state. The 2*A*_g_ state is responsible for almost all the observed DPO fluorescence and gains oscillator strength via vibronic intensity stealing with the near-degenerate 1*B*_u_ state. The fluorescence lifetime associated with the 2*A*_g_ state decayed monoexponentially (4.2–7.2 ns) in contrast to prior biexponential decay kinetics reported for similar polyenes, diphenylbutadiene and diphenylhexatriene. An analysis combining the measured fluorescence lifetimes and fluorescence quantum yields (the latter varying between 7 and 21%) allowed for a 190 cm^–1^ Herzberg–Teller vibronic coupling constant between the 1*B*_u_ and 2*A*_g_ states to be determined. The analysis also revealed that the ordering of electronic states remains constant in all the solvents studied, with the 2*A*_g_ state minimum always lower in energy than that of the 1*B*_u_ state, thus making it a relatively simple polyene compared to structurally similar diphenylhexatriene.

## 1 Introduction

**Figure 1:**
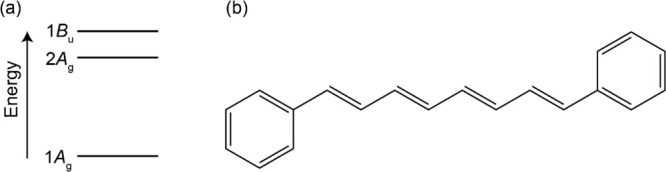
(a) Schematic energy level structure and (b) chemical structure of diphenyloctatetraene (DPO).

Polyenes are an important class of π-conjugated molecules which play a crucial dual role in photosynthesis, acting as accessory light-harvesting pigments and photoprotective radical quenchers.^1−6^ Despite several decades of spectroscopic study, the photophysics of polyenes continue to be popular, in part, because their complex electronic structure is still highly debated and varies strongly with chain length, chemical substitution, and environment.^1−3,7−11^ The low-energy photophysics of many polyenes is governed by the interconversion between two excited electronic states with different symmetries in the *C*_2*h*_ point group.^2,12−14^ The electronic ground state of polyenes has *A*_g_ symmetry (corresponding to the 1*A*_g_ state), and the lowest energy optically “bright” electronic state has *B*_u_ symmetry (1*B*_u_ state).^2,15,16^ Early seminal work by Hudson and Kohler^13^ proposed that a low-lying electronic state of *A*_g_ symmetry (henceforth referred to as the 2*A*_g_ state) is also important but is formally electrically dipole forbidden and thus termed optically “dark” (see Figure 1a).^11,17−23^

The excitation energies associated with the 2*A*_g_ and 1*B*_u_ states are stabilized as the number of double bonds (*N*) in the polyene backbone increases.^3,23−25^ However, due to the differences in electronic character, the stabilization energy per double bond is greater for the 2*A*_g_ state. As a result, for shorter polyenes with conjugation lengths of ∼*N* ≤ 2, such as diphenylbutadiene, the prevailing consensus is that the 1*B*_u_ bright electronic state is the lowest energy singlet state.^26−28^ The relative ordering of the first two excited states in the vertical Franck–Condon region is thought to invert at *N* ∼3–4—see Figure 1a. For “long” polyenes (*N* ≥ 5), such as photosynthetic carotenoids, the 2*A*_g_ state is the lowest energy singlet excited state^1−3,13,23^ and is populated by rapid internal conversion from the photoexcited 1*B*_u_ state.

The situation is less clear for polyenes with intermediate conjugation lengths (*N* ∼3–4), where it is expected that the 1*B*_u_ and 2*A*_g_ state energies will be energetically close,^24,25^ e.g., for diphenylpolyenes such as diphenylhexatriene (DPH) and diphenyloctatetraene (DPO). DPH has received considerable attention due to its rich photophysics,^10,11,17−19,22,29−32^ use as a fluorescent sensor,^33,34^ and utilization in solar energy applications.^35−38^ There are competing reports of the excited state dynamics of DPH.^10,22,29^ It has been proposed that absorption and fluorescence do not occur from the same state in DPH.^10^ While absorption occurs to the strongly allowed 1*B*_u_ state, fluorescence occurs on a nanosecond time scale (with appreciable quantum yields) from the formally optically dark 2*A*_g_ state. The latter state gains oscillator strength via Herzberg–Teller vibronic intensity borrowing from the strongly allowed 1*B*_u_ state.^39^ In this proposed model, vibrations of *b*_*u*_ symmetry distort the molecular structure, relaxing the symmetry requirements and allowing the 2*A*_g_ state to fluoresce. The above proposed model for DPH is still debated, with one hypothesis that the 2*A*_g_ state is not involved in the excited state deactivation,^22^ and the large observed Stokes shift and lack of mirror symmetry between absorption and fluorescence can be rationalized by a significant excited state geometric rearrangement.

Conversely, the photophysics of DPO, which has an additional double bond along the polyene backbone (chemical structure given in Figure 1b), has been far less intensively studied than DPH, with only a few studies to date examining the sub-nanosecond dynamics,^31,40−42^ the fluorescence quantum yield,^43,44^ and the nanosecond kinetics^18,41,44^ in only weakly interacting solvents. Given the longer chain length, it is anticipated that the 1*B*_u_–2*A*_g_ energy gap will be larger in DPO than DPH and strongly influence the excited state photophysics and associated fluorescence quantum yield. To comprehensively investigate the photophysical dynamics of DPO, steady-state and wavelength-resolved fluorescence lifetime spectroscopies were used to investigate how the dynamics varied in 10 solvents of different polarities and polarizabilities, e.g., ranging from weakly interacting solvents such as cyclohexane to polar hydrogen-bonding solvents such as methanol.

## 2 Experimental and Computational Methods

### 2.1 Sample Preparation and Steady-State Spectroscopy

DPO (99%) was purchased from BOC Sciences and used without any further purification. DPO was dissolved in 10 different solvents purchased from Sigma-Aldrich (>99% purity). For fluorescence quantum yield and wavelength-resolved time-correlated single photon counting (WR-TCSPC) measurements, the solutions were diluted to have an absorbance of ∼0.1 and ∼0.3 at the peak wavelength of the pump laser in a static 1 cm path length cuvette, respectively. Each sample was freshly prepared before ultrafast measurements. The fluorescence quantum yield of DPO in the various solvents was determined using an Edinburgh Instruments FS5 fluorimeter and SC-30 integrating sphere.

### 2.2 Wavelength-Resolved Time-Correlated Single Photon Counting Measurements

WR-TCSPC measurements were acquired using a modified version of the home-built TCSPC apparatus previously reported.^45^ The output of a tunable high-power ultrafast oscillator (Chameleon Ultra II, 3.7 W, 80 MHz, Coherent) was frequency doubled to generate the required UV excitation pulses (392–408 nm). To avoid re-excitation of samples, the repetition rate was reduced to 6.67 MHz using a pulse picker (APE cavity dumper). The diffracted output was spatially filtered with a pinhole to remove residual zeroth order diffraction, and the resulting <15 pJ (per pulse) UV light was focused into a static liquid sample (1 cm path length cell). Fluorescence was collected at 90° relative to excitation using an infinity-corrected microscope objective (4×/0.2 NA Plan Apochromat, Nikon) and through a series of achromatic lenses collimated and focused onto an avalanche photodiode detector (ID100-50-ULN, IDQ).

Photon count arrival times were recorded with a time-to-digital converter (Time Tagger 20, Swabian Instruments) and accumulated in 10 ps bins. To facilitate wavelength-resolved TCSPC measurements, fluorescence was propagated through a birefringent interferometer (Gemini, Nireos)^46^ after the collection objective and before the detector. Each data point was integrated for 1 s, and every spectral interferogram was averaged 10 times. All WR-TCSPC data were acquired by using customized LabVIEW software (National Instruments). Spectral filtering due to transmission through the collecting objective and Gemini interferometer was corrected for in postprocessing. The instrument response function (IRF) was 170 ps, as determined by recording laser scatter from solvent in a 1 cm path length cuvette. The wavelength dependence characterization of the IRF is given in the Supporting Information (see Figure S4). All data were collected at room temperature (20 °C) using the magic angle condition.

### 2.3 Density Functional Theory Calculations

Density functional theory (DFT) and time-dependent DFT (TD-DFT) calculations were performed using the Gaussian 16 computational suite.^47^ The minimum energy geometries of the ground electronic state and optically bright excited state were calculated by using the ωB97-XD exchange-correlation density functional and the def2-SVP basis set. Solvation effects were included in vertical excitation energy calculations using a polarizable continuum model.

## 3 Results and Discussion

### 3.1 Steady-State Spectroscopy

**Figure 2:**
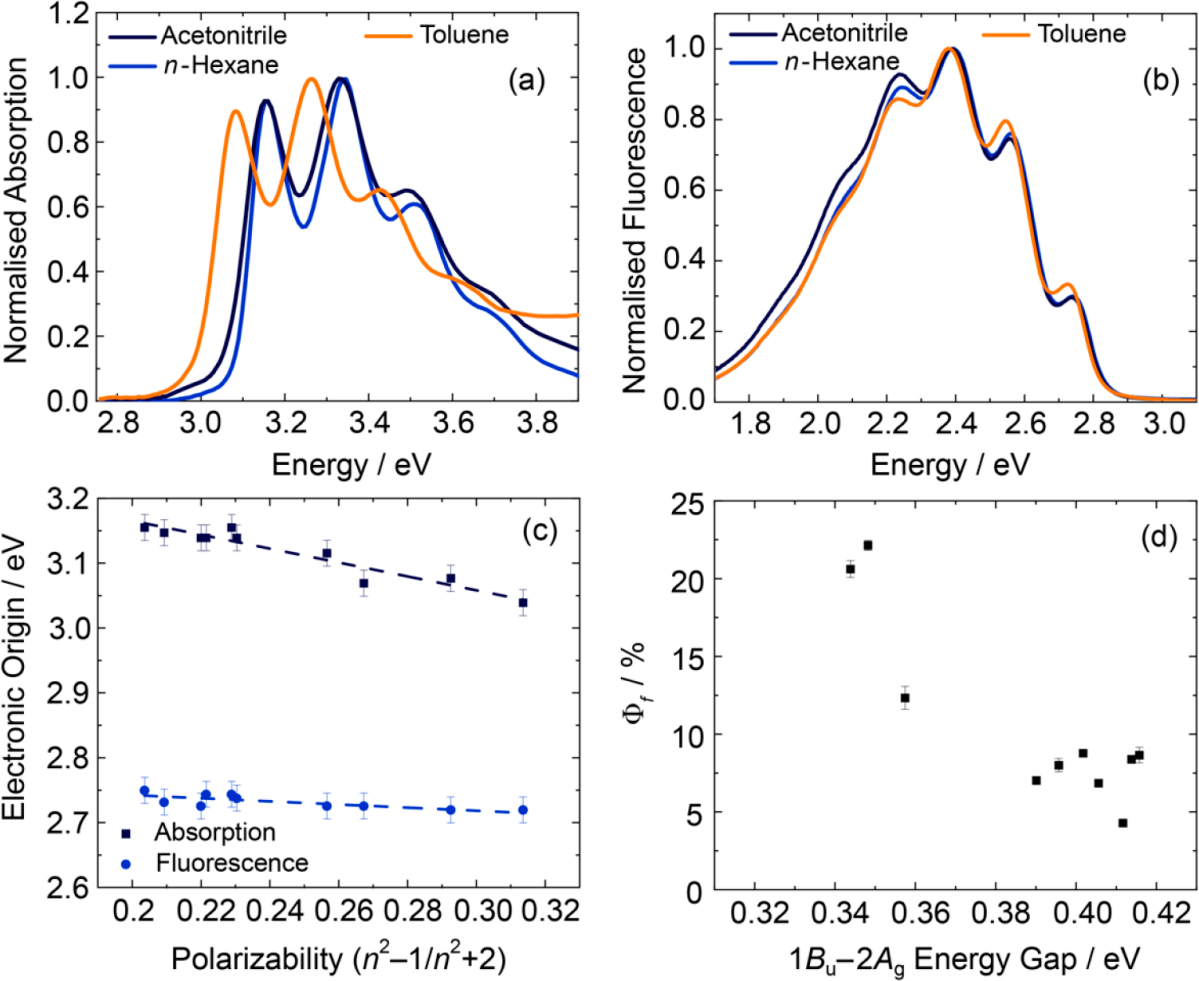
Normalized steady-state (a) absorption and (b) fluorescence spectra of DPO in three different solvents. (c) Absorption and fluorescence electronic origins derived from steady-state spectra as a function of solvent polarizability, (*n*^2^ – 1)/(*n*^2^ + 2), where *n* is the refractive index of the solvent. (d) Experimentally determined fluorescence quantum yield (Φ_f_) of DPO as a function of the estimated 1*B*_u_–2*A*_g_ energy gap.

The steady-state absorption and fluorescence spectra of DPO in acetonitrile, *n*-hexane, and toluene are shown in Figure 2a,b. Focusing first on the solvent-dependent absorption spectra (Figure 2a), the absorption spectra shift to lower energy with increasing solvent polarizability, however, seemingly without any major change in line shape. Making the reasonable assumption that the lowest energy vibronic peak in these data corresponds to the 1*B*_u_ electronic origin (0–0 band), a linear trend was found as a function of the solvent polarizability (α, commonly modeled as (*n*^2^ – 1)/(*n*^2^ + 2)),^48^ as shown in Figure 2c, with similar trends reported for many polyenes previously.^11,23,49,50^

The polarization-dependent excitation energies take the form of a linear slope^10^
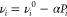
1where *ν*_*i*_ and ν_*i*_^0^ are the solution phase and gas phase excitation energies associated with the *i*th electronic state. The gradient, *P*_*i*_, is related to the associated electronic transition dipole moment of the electronic state, and thus, higher oscillator strength transitions are expected to exhibit greater polarizability-dependent solvatochromic shifts.

Fitting the data in Figure 2c to eq 1 results in gradients of 2000 ± 500 and 9000 ± 1000 cm^–1^ for the 2*A*_g_ and 1*B*_u_ states, respectively. Extrapolation of these fits to zero polarizability allows the gas phase energy gap between the two states to be estimated as 4600 ± 2200 cm^–1^. These values are used to calculate the differential solvation and energy gap of the two electronic states in section 3.2.

To investigate whether the low-energy portion of the absorption spectra (2.8–3.5 eV) is dominated by vibronic transitions to a single electronic state, the experimental absorption spectra were modeled using a Franck–Condon progression for a single harmonic oscillator.^51,52^ Full details, including the limitations of the model, are given in section 1 of the Supporting Information. Using this model, experimental spectra were reproduced with excellent agreement using a single Franck–Condon active vibrational wavenumber of 1520 ± 10 cm^–1^ most likely corresponding to a C=C stretching nuclear mode^12,53,54^ and an associated Huang–Rhys factor (*S*) of 1.27. Across the different solvents, only the 0–0 band energy was altered in the modeled fit to capture the solvatochromic shift. The success of the displaced harmonic oscillator model to reproduce the experimental absorption spectra confirms that long-wavelength absorption is dominated by, or solely attributable to, a single electronic transition in all 10 solvents.

To support the experimental observations, density functional theory (DFT) and time-dependent (TD)-DFT calculations were conducted on the 1*A*_g_ and 1*B*_u_ electronic states initially in the gas phase. The 2*A*_g_ state was not investigated, as TD-DFT is unable to properly capture the expected doubly excited nature of this transition.^55,56^ Geometry optimization of the ground state with DFT returned a planar geometry—the associated structure and bond lengths are shown in Figure S3a, displaying a strong alternation of the C=C (1.35 Å) and C—C (1.45 Å) bond lengths along the DPO backbone, in line with prior computational studies of polyenes.^25,57,58^ TD-DFT vertical excitation calculations predict that the 1*B*_u_ state has a very large oscillator strength (*f* = 2.567). TD-DFT optimization of the 1*B*_u_ excited state returned a planar geometry, with near identical bond lengths (1.40 Å, Figure S2a) associated with the central linear carbon chain of DPO, and some minor alternation toward the terminal phenyl rings (1.39/1.40 Å), in accord with prior findings.^25,57,58^ The solvatochromic shift in the 1*B*_u_ origin observed experimentally was modeled in TD-DFT calculations by the inclusion of a polarizable continuum solvent model in the vertical excitation energies. The results of these calculations are shown overlaid with experimental results in Figure S3 and yield a quantitatively similar trend.

The fluorescence spectra for DPO in *n*-hexane, toluene, and acetonitrile are shown in Figure 2b. Compared to the respective absorption data (Figure 2a), the fluorescence spectra do not mirror the absorption spectral line shape and also fail to exhibit the same strong polarizability-dependent solvatochromic shift (Figure 2c). The fluorescence spectra were successfully modeled using the same approach outlined above for absorption, with a Franck–Condon progression in a single vibrational mode of wavenumber 1390 ± 10 cm^–1^, with *S* varying between 2.27 and 2.45 across the 10 solvents (<10%). Such a variation in the Huang–Rhys factor is likely an indicator of small changes in displacement of the fluorescent state potential energy minima.^51,59,60^ The ability of the model to reproduce the fluorescence profiles indicates that the fluorescence originates primarily from a single excited state. The lack of mirror symmetry between absorption and fluorescence spectra and different solvatochromic behavior supports the hypothesis that fluorescence occurs from a different electronic state than initially excited. Therefore, in agreement with other studies,^10,18,40^ DPO fluorescence dominantly originates from the 2*A*_g_ state. Prior studies have reported very weak additional bands in fluorescence spectra (at shorter wavelengths than the main band) associated with the 1*B*_u_ state;^40,61^ however, these were not observed in the present study.

As determined above, the vast majority of DPO fluorescence originates from the formally dipole forbidden 2*A*_g_ state and, therefore like other polyenes, must gain oscillator strength via Herzberg–Teller intensity borrowing from the proximal optically bright 1*B*_u_ state. For this mechanism to be effective, it requires strong coupling between the bright and dark electronically excited states, which is directly dependent on their relative minimum energies.^10^ Due to the differential solvent stabilization of the two lowest energy excited states of DPO, the 1*B*_u_–2*A*_g_ energy gap can be controlled by small changes in the solvent polarizability.

The DPO fluorescence quantum yield as a function of the estimated 1*B*_u_–2*A*_g_ state energy gap is displayed in Figure 2d, and a negative trend is observed. Overall, the fluorescence quantum yield varies between 4.3 and 21.2%, with a mean of 5.7% and standard deviation of 10.6%. However, as the fluorescence yield is not close to unity, there must be competitive non-radiative excited state deactivation pathways, which may also vary as a function of solvent and affect the fluorescence quantum yield. To further investigate the excited state dynamics of DPO, wavelength-resolved time-correlated single photon counting (WR-TCSPC) measurements were undertaken.

### 3.2 WR-TCSPC

**Figure 3:**
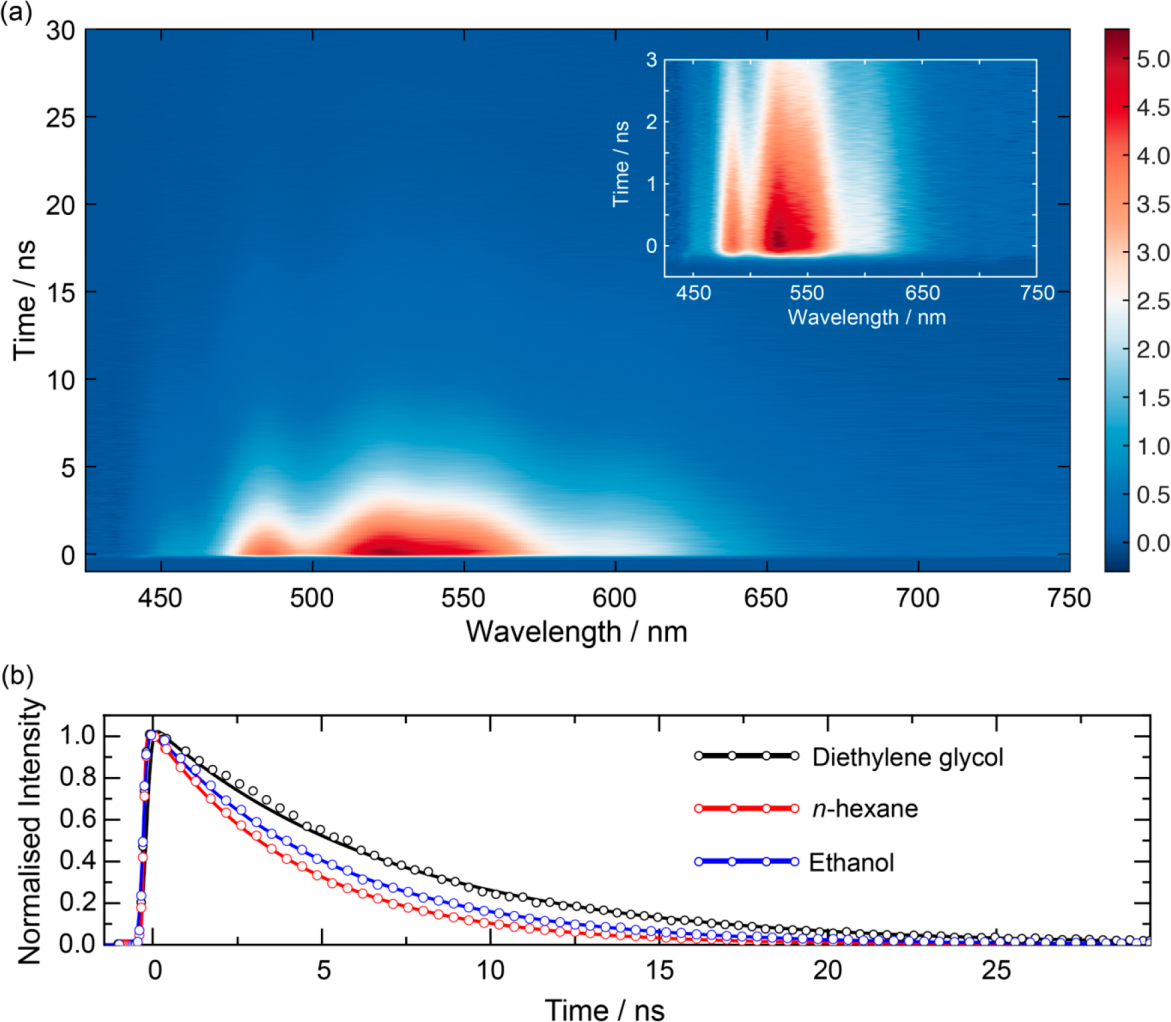
(a) WR-TCPSC false color contour map of DPO in *n*-hexane. The inset shows the first few nanoseconds of the fluorescence decay. (b) Example fluorescence decay kinetics (dots, sampled every 400 ps) for DPO in room temperature ethanol, *n*-hexane, and diethylene glycol and overlaid fits (solid lines).

The wavelength-resolved time-correlated fluorescence spectrum of DPO in *n*-hexane is displayed in Figure 3a. There were no apparent shifts in the fluorescence line shape observed outside of the IRF in any solvent, and the fluorescence intensity decayed monotonically. Spectral slices for several different time delays are shown in Figure S5 for DPO dissolved in *n*-hexane and ethanol. Figure 3b shows representative kinetics obtained by averaging over the entire emission band for three solvents. Overlaid are fits to the data modeled as a monoexponential decay convolved with a Gaussian IRF function. The time constants returned from fitting to the experimental time-resolved fluorescence data are listed in Table 1.

**Table 1:** Experimentally Determined DPO Fluorescence Quantum Yield (Φ_f_), Fluorescence Lifetime (τ_f_), Derived Fluorescence Rate Constant (*k*_f_), Absorption, and Fluorescence Origins in 10 Different Solvents

Solvent	(*n*^2^ – 1)/(*n*^2^ + 2)	Φ_f_/%	τ_f_/ns	*k*_f_/×10^7^ s^–1^	Absorption origin/eV	Fluorescence origin/eV
methanol	0.204	6.9 ± 0.2	5.07 ± 0.17	1.35 ± 0.05	3.16 ± 0.02	2.75 ± 0.02
acetonitrile	0.209	8.7 ± 0.5	5.13 ± 0.17	1.69 ± 0.06	3.15 ± 0.02	2.73 ± 0.02
acetone	0.220	8.4 ± 0.2	5.11 ± 0.17	1.64 ± 0.05	3.14 ± 0.02	2.73 ± 0.02
ethanol	0.221	8.0 ± 0.5	5.38 ± 0.17	1.49 ± 0.05	3.14 ± 0.02	2.74 ± 0.02
*n*-hexane	0.229	4.3 ± 0.1	4.29 ± 0.17	0.99 ± 0.04	3.16 ± 0.02	2.74 ± 0.02
2-propanol	0.230	8.8 ± 0.2	5.19 ± 0.17	1.69 ± 0.06	3.14 ± 0.02	2.74 ± 0.02
cyclohexane	0.257	7.0 ± 0.1	5.36 ± 0.17	1.31 ± 0.04	3.12 ± 0.02	2.73 ± 0.02
diethylene glycol	0.267	20.6 ± 0.5	7.21 ± 0.17	2.86 ± 0.07	3.07 ± 0.02	2.73 ± 0.02
toluene	0.293	12.3 ± 0.7	4.87 ± 0.17	2.54 ± 0.09	3.08 ± 0.02	2.72 ± 0.02
benzyl alcohol	0.314	21.2 ± 1.4	6.53 ± 0.17	3.25 ± 0.08	3.04 ± 0.02	2.72 ± 0.02

The lack of a wavelength dependence on the fluorescence decay kinetics measured in all solvents is in agreement with steady-state measurements and indicates that all the observed fluorescence originates from the 2*A*_g_ state. This necessitates that the internal conversion transfer from the 1*B*_u_ state must be rapid and complete in a time scale shorter than the instrumental response (170 ps). Previous studies disagree on the time scale for 1*B*_u_ → 2*A*_g_ interconversion in DPO reporting time constants of <1 ps and hundreds of ps.^31,40−42^ Our results are in accord with the prompter interconversion time scale.

The time-resolved fluorescence data are dominated by a monoexponential decay, with solvent-dependent nanosecond lifetimes, which are presented in Table 1. There is a significant variation in fluorescence lifetime, which falls within the range 4.29–7.21 ns, and has a mean value of 5.41 ns and 0.84 ns standard deviation. The monoexponential fluorescence decay observed for DPO contrasts with the biexponential nanosecond fluorescence dynamics previously observed in similar chain length polyenes such as diphenylbutadiene (DPB)^62^ and DPH,^63^ indicating far simpler dynamics in this longer chain diphenylpolyene.

Rather than analyzing the fluorescence lifetimes, these data were converted into fluorescence rates (*k*_f_) using the fluorescence quantum yield and fluorescence lifetime as defined in eqs 2 and 3
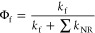
2
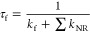
3which via substitution yields
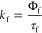
4where Φ_f_ is the fluorescence quantum yield, Σ*k*_NR_ is the sum of non-radiative rate constants, and τ_f_ is the fluorescence lifetime derived from fits to WR-TCSPC data.

Andrews and Hudson,^10^ following the work of Hug and Becker,^64^ derived an expression for the fluorescence rate of a forbidden electronic state mediated by vibronic intensity borrowing based on perturbation theory—an adapted form is given in eq 5
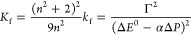
5where *K*_f_ is the radiative rate of the formally dipole forbidden state corrected for the local field; e.g., in the gas phase, α, *n*, and *k*_f_ are defined earlier. The remaining three parameters in eq 5 are variables: Δ*E*^0^ corresponds to the energy gap between the 1*B*_u_ and 2*A*_g_ states in the gas phase, Δ*P* is the differential solvation between the two electronic states, and both quantities can be estimated as discussed in section 3.1. Γ^2^ is a collection of molecular specific terms:
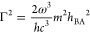
6In eq 6, ω is the central fluorescence wavelength, *h* is Planck’s constant, *c* is the speed of light, *m* is the transition dipole moment of the 1*B*_u_ state, and *h*_BA_ is the vibronic coupling term between the optically bright and dark states.

Andrews and Hudson’s model^10^ presumed the solvent has no direct effect on the solute and would thus be unable to promote non-totally symmetric distortions. This approximation is most appropriate for non-polar solvents with low permanent dipole moments. In polar solvents, the effect of the solvent permanent dipole in the immediate region surrounding the solute will have a more significant effect. This instantaneous interaction will promote greater non-totally symmetric distortions in the solute which are not a result of vibronic mixing. This effect is solely due to direct interactions with the solvent and is independent of the solvent polarizability. As a result, it is expected that the radiative rates determined for polar solvents (with higher dielectric constants) will be greater than those in non-polar solvents due to the non-totally symmetric distortions in the DPO nuclear framework generated by the solvent.

**Figure 4:**
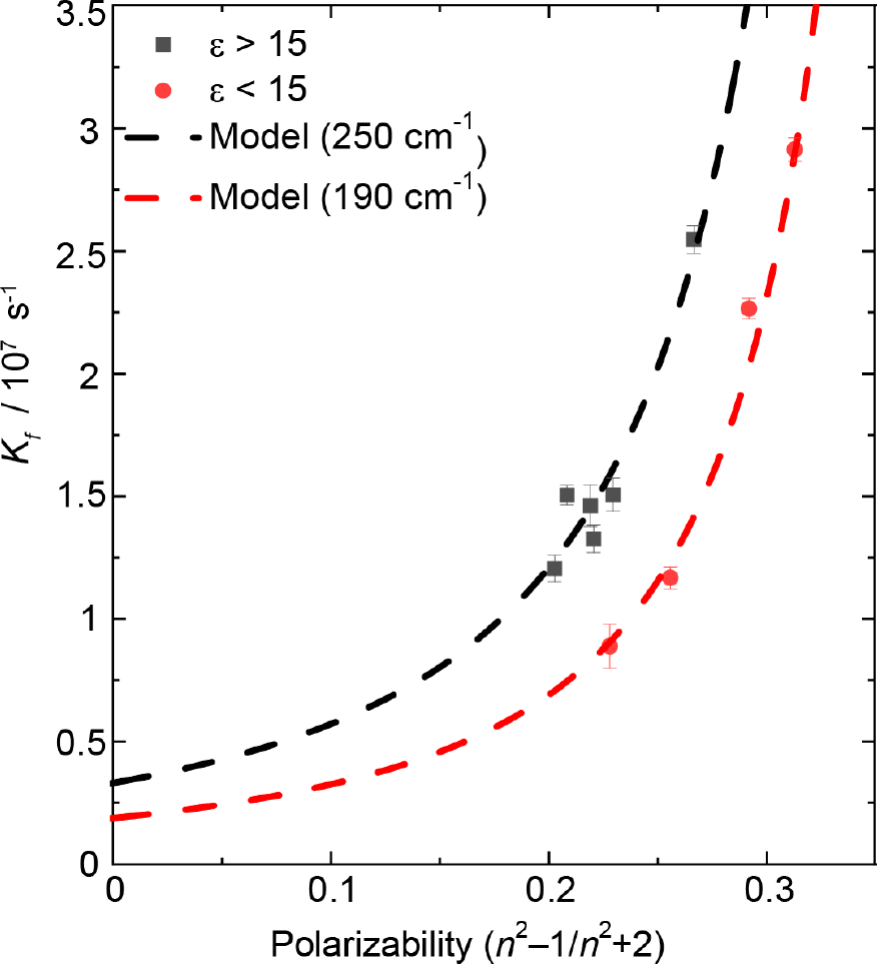
Measured radiative rate corrected for local solvent effects as a function of the solvent polarizability. Overlaid dashed lines correspond to the results of the intensity borrowing model discussed in the main text for the two solvent dielectric regimes.

All measurements were acquired in solution and thus were corrected for the local solvent field (*n*^2^ + 2)^2^/9*n*^2^, as derived previously.^65^Figure 4 shows the corrected fluorescence rates (*K*_f_) as a function of solvent polarizability, with close to quadratic behavior evident. Two trends were evident for solvents with high (ε < 15) and low (ε > 15) dielectric constants.

Evaluation of the model outlined in eq 5 yielded a good agreement with the experimental data; see overlaid models with experimental data in Figure 4. The modeling returns three quantities, Δ*E*^0^, Γ^2^, and *h*_BA_. To obtain a good fit to data, Δ*E*^0^ was allowed to vary within the range determined in section 3.1, with 3350 cm^–1^ returned from modeling. This value is in reasonable agreement with gas phase measurements of DPO which estimated the 1*B*_u_–2*A*_g_ energetic gap to be ∼4000 cm^–1^.^66^ The differential solvation parameter returned from the evaluation of eq 5 gave a value of 8000 cm^–1^, which is within the bounds determined in section 3.1 (7000 ± 1000 cm^–1^). Finally, from the returned value of Γ^2^ it is possible to extract a vibronic coupling constant using eq 6 and the 1*B*_u_ ← 1*A*_g_ transition dipole moment calculated using TD-DFT (13.75 D). Using this method, *h*_BA_ values were estimated to be 250 and 190 cm^–1^ for high and low polarity solvents, respectively. This value is reasonable for intensity borrowing (∼hundreds of cm^–1^)^10^ and is consistent with those previously calculated for other polyenes—DPH and retinol are estimated to have vibronic coupling constants of 555 and 149 cm^–1^, respectively.^10^ Further, as expected, the high polarity data requires a larger coupling constant likely due to the increased non-totally symmetric vibrations induced by interactions with the solvent, which are not fully accounted for in the simplistic static term used to correct for the local field. Therefore, the 190 cm^–1^ coupling constant obtained from solvents of low dielectric constants is the most reliable value determined. The success of Andrews and Hudson’s model is strong evidence to support the hypothesis that the vast majority of DPO fluorescence originates from the 2*A*_g_ state and that the electronic state gains oscillator strength via vibronic intensity stealing from the 1*B*_u_ state.

There are important parallels and differences between the observed photophysics of DPO revealed in the present study and the prior investigations of related polyene DPH. The fluorescence quantum yield for DPO varies between 4.3 and 21.2% and increases as a function of the solvent polarizability (Table 1). The room temperature fluorescence quantum yield of DPH has only been recorded in a smaller range of solvents such as saturated alkanes where the channel is far more important (62–65%) than in DPO. However, in less polarizable and polar solvents such as ethanol and acetonitrile the fluorescence quantum yield of DPH (15–25%)^11^ is similar to the maximum fluorescence yield in DPO.

Regardless of the solvent, there is a large percentage of excited DPO (and DPH) molecules that do not relax via fluorescence, leaving much of the excited state dynamics unaccounted for. An intersystem crossing pathway can be excluded as prior photosensitization experiments reported triplet quantum yields for DPH and DPO of <1% in conventional polar and non-polar solvents.^67^ Therefore, photoexcited molecules that do not fluoresce must either internally convert directly to the electronic ground state from the 2*A*_g_^2^ or 1*B*_u_ states^68^ or alternatively photoisomerize as reported for DPO, DPH, and also DPB.^43^ The quantum yield for *trans* → *cis* photoisomerization of DPH is 36% in acetonitrile, a solvent with a correspondingly modest (25%) fluorescence quantum yield.^29^ The photoisomerization yields have, to the best of our knowledge, not been reported for DPO, but given the structural similarity with DPH we speculate that this must also be a key non-radiative excited state decay channel for DPO in all solvents given the fairly low (<22%) fluorescence quantum yield.

One important factor that will determine whether fluorescence is the most competitive decay pathway for the 2*A*_g_ state of diphenylpolyenes compared to other non-radiative pathways is the radiative rate, which for the formally dipole forbidden electronic transition that gains oscillator strength via intensity borrowing is inversely proportional to [Δ*E*^0^(1*B*_u_–2*A*_g_)]^2^—see eq 5. We have determined Δ*E*^0^ for DPO (via extrapolation from steady-state data) to be 4600 cm^–1^, which is ∼1/3 greater than the energy gap for DPH (3410 cm^–1^).^32^ The differences in Δ*E*^0^ help to reconcile the 2–3× greater radiative rates reported for DPH^11^ compared to those determined here for DPO.

## 4 Conclusions

The photophysics of DPO have been comprehensively studied, in a far wider range of solvents than previously investigated, ranging from weakly interacting non-polar solvents to hydrogen-bonding alcohols. These studies utilized steady-state absorption and fluorescence spectroscopies alongside wavelength-resolved time-correlated single photon counting measurements to establish a more detailed model for the excited state dynamics of DPO than hitherto reported. These data reveal that photoexcitation to the 1*B*_u_ state of DPO leads to fast (<170 ps) internal conversion to the lower-lying optically dark 2*A*_g_ state in agreement with some prior studies.^31,41,42^ The 2*A*_g_ state is responsible for the vast majority of fluorescence and gains oscillator strength via intensity borrowing from the optically bright 1*B*_u_ state. The Herzberg–Teller vibronic coupling constant between the 1*B*_u_ and 2*A*_g_ states was estimated to be 190 cm^–1^. The wavelength-resolved time-dependent fluorescence data show no spectral evolution within our time resolution but reveal that the 2*A*_g_ state fluoresces back to the ground state monotonically, in contrast to prior observations of DPH and DPB which exhibit biphasic fluorescence decay from the lowest excited singlet state.^26,63^ These studies demonstrate that the electronic state ordering must be conserved in all of the solvents studied, and the 2*A*_g_ minimum is always lower in energy than that of the corresponding 1*B*_u_ state. The maximum fluorescence quantum yield was found to be 21% in benzyl alcohol solution—the most polarizable solvent studied. As a result, this means the majority of photoexcited DPO molecules decay from the excited state via alternative non-radiative decay pathways including *trans* → *cis* photoisomerization and potentially direct internal conversion to the ground electronic state. As evident from the data presented, the fluorescence rate and quantum yield of DPO are very sensitive, in a predictable manner, to the refractive index of the environment, due to the photophysical model proposed. The net result is the environmental sensitivity of DPO, utilized for *in vivo* fluorescent bioimaging, can be rationalized with fundamental molecular photophysics.

## Supporting Information Available

Franck–Condon factor modeling; TCSPC instrument response function; slices of wavelength-resolved TCSPC data; minimum energy DFT and TD-DFT structures; and calculated and experimental 1*B*_u_ excitation energies (PDF)

## Notes

Data are available from the University of Bristol data repository at https://doi.org/10.5523/bris.1qq9ffnrwc5gl2jc27ip9jog75.
