# Supplementary material for: The Solvent-Dependent Photophysics of Diphenyloctatetraene
Source: J Phys Chem B. 2023 Sep 14;127(38):8199–207. doi: 10.1021/acs.jpcb.3c03737 (PMC10544004; doi:10.1021/acs.jpcb.3c03737)
Supplement: Supplementary file 1 — jp3c03737_si_001.pdf [file jp3c03737_si_001.pdf]

The Solvent Dependent Photophysics of Diphenyloctatetraene

Daniel W. Polak,[‡] Alexandros D. P. Hannon, Guilherme A. Marczak Giorio, Olivia A. Hawkins and Thomas A. A. Oliver*

School of Chemistry, Cantock's Close, University of Bristol, Bristol, BS8 1TS, UK

[‡] Current address: Department of Chemistry, University of Leicester, Leicester LE1 7RH, UK

*Author for correspondence: tom.oliver@bristol.ac.uk

1. Franck-Condon Modelling

The vibronic structure within experimental absorption and fluorescence of DPO in solution were modelled using a simple displaced harmonic oscillator Franck-Condon model, as previously outlined in refs. [1,2]. The model relies on the following assumptions: (i) electronic transitions couple to a single dominant vibrational mode; (ii) all vibronic peaks have the same full-width half-maximum; (iii) the associated line shape for all peaks is the same and Gaussian; (iv) the Franck-Condon progression ignores effects of anharmonicity.

The intensity of the vibronic progressions should follow a Poisson distribution, as described by Equations S1 and S2 for fluorescence and absorption respectively:

$$I_{Fl}(E) = (n_E E)^3\, \Sigma_m \left(\mathrm{e}^{-S} \frac{S^m}{m!}\, \mathrm{e}^{-\left(\frac{(E-(E_0-m\omega_{\mathrm{vib}}))^2}{\sigma^2}\right)} \right) \tag{S1}$$

$$I_{Abs}(E) = (n_E E)\, \Sigma_m \left(\mathrm{e}^{-S} \frac{S^m}{m!}\, \mathrm{e}^{-\left(\frac{(E-(E_0+m\omega_{\mathrm{vib}}))^2}{\sigma^2}\right)} \right) \tag{S2}$$

where, $I_{Fl}(E)$ and $I_{Abs}(E)$ are the intensity of the fluorescence and absorption as a function of energy E (in eV), and n_E is the energy dependent refractive index. ω_{vib} is the associated energy with the Franck-Condon active vibrational mode and S the associated Huang-Rhys factor, m is the vibrational quantum number, E_0 is the electronic origin associated with m = 0, and σ determines the Gaussian linewidth of each vibrational peak, as defined by the full-width half-maximum = $2\sigma\sqrt{2\ln 2}$.

Modelled absorption and fluorescence spectra for DPO in cyclohexane (overlaid with experimental data) are shown in Figure S1. The same parameters, S = 1.27, ω_{vib} = 0.188 ± 0.001 eV (1520 ± 10 $\mathrm{cm^{-1}}$) and σ = 140 meV, were used to model the absorption spectra of DPO in all 10 different solvents, only altering E_0, which is derived from experimental data (see Table

1 in main manuscript). For fluorescence spectra, good agreement with experimental data was achieved using ω_{vib} = 0.172 ± 0.001 eV (1390 ± 10 cm^{-1}) and σ =162 meV. The electronic origin was experimentally derived, however, unlike absorption spectra, the Huang-Rhys factor for fluorescence varied between 2.27 and 2.45 between different solvents, as expected for fluorescence intensity derived from a Herzberg-Teller intensity borrowing mechanism.

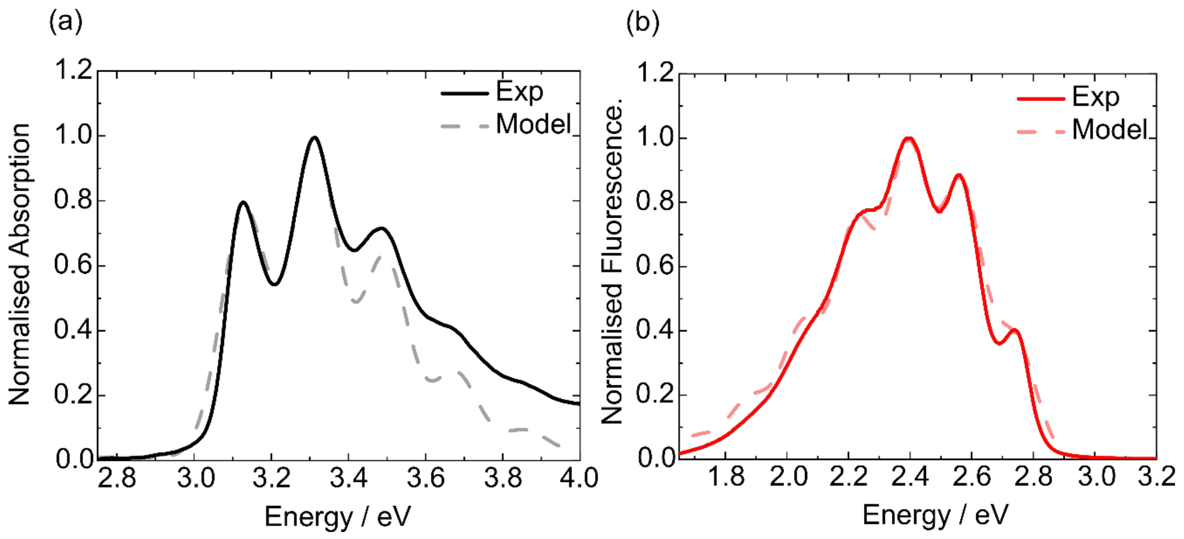

Figure S1 Comparison of steady state normalised (a) absorption and (b) fluorescence data of DPO in cyclohexane with Franck-Condon model (dashed line) of data. Details of parameters used are given in the text.

2. DFT and TD-DFT calculated minimum energy structures

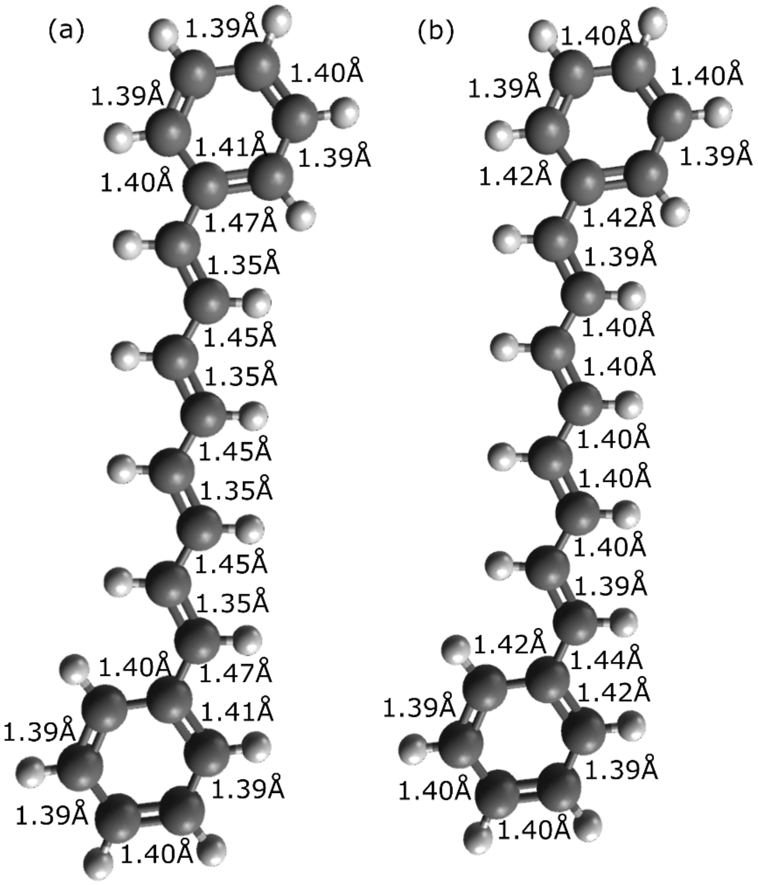

Figure S2 Minimum energy geometries of the (a) S_0 and (b) $1B_u$ states of DPO. Bond lengths associated with the polyene chain and terminal phenyl rings are overlaid.

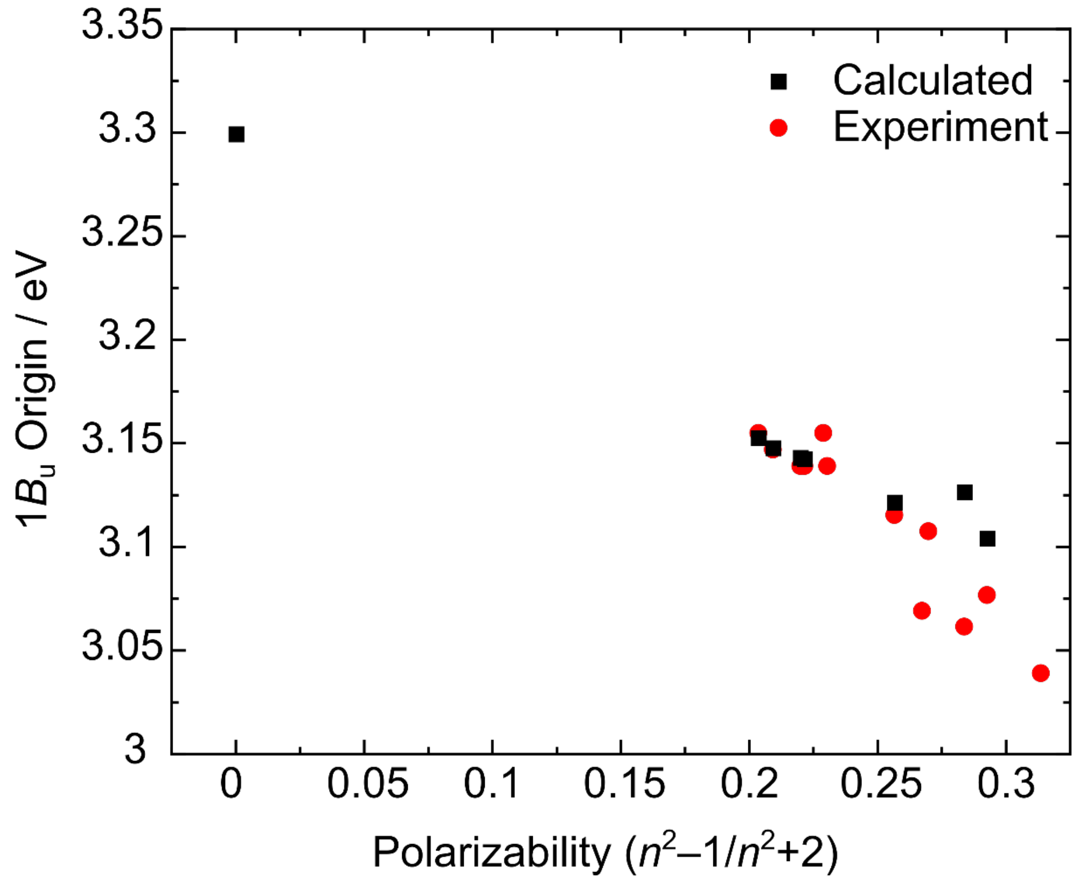

Figure S3 Comparison of experimental and calculated vertical $1B_u$ excitation energies as a function of solvent polarizability. The calculated excitation energies have been shifted by –160 meV to aid comparison.

3. Instrument Response Function

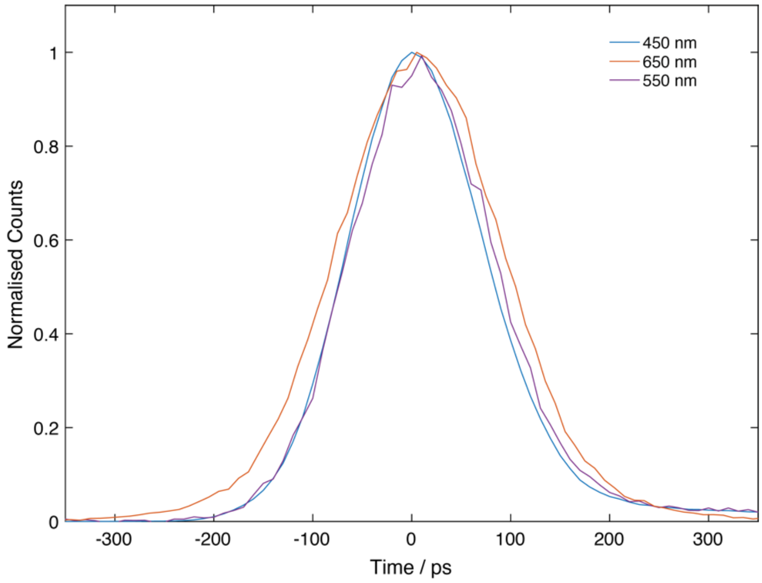

Figure S4 Instrument response function measured by solvent scatter for three displayed laser excitation wavelengths.

4. WR-TCSPC Spectra

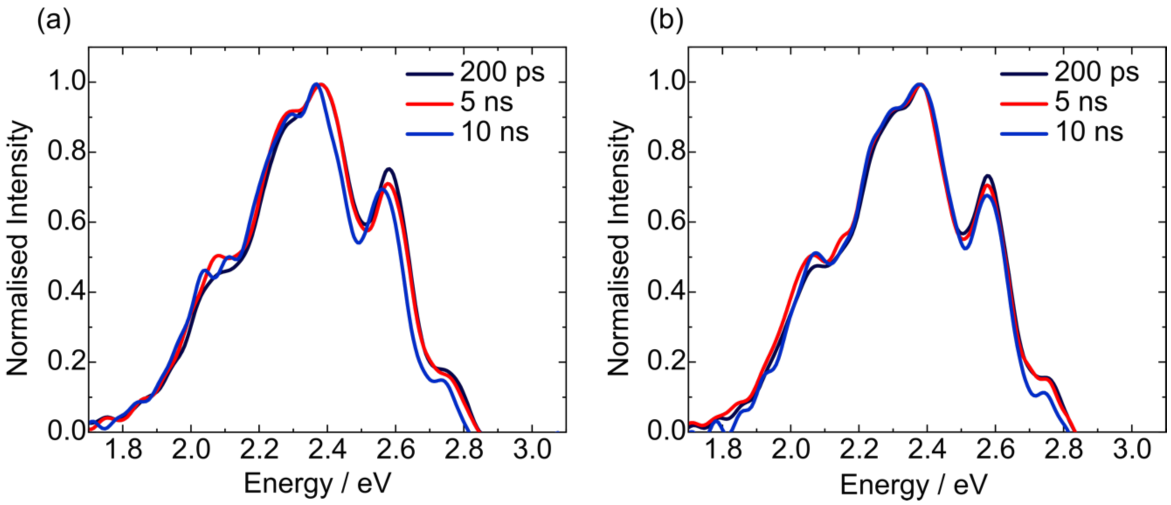

Figure S5 Normalised fluorescence spectra averaged over 200 ps windows from WR-TCSPC measurements of DPO in (a) *n*-hexane and (b) ethanol for a series of delays shown in the legend.

References

(1) Köhler, A.; Bässler, H. *Electronic Processes in Organic Semiconductors*; Wiley-VCH Verlag GmbH & Co. KGaA: Weinheim, 2015.

(2) Saidani, M. A.; Benfredj, A.; Hamed, Z. B.; Romdhane, S.; Ulbricht, C.; Egbe, D. A. M.; Bouchriha, H. Franck-Condon Analysis of the Photoluminescence Spectra of a Triple-Bond Containing Polymer as a Solution and as a Thin Film. *Synth. Met.* **2013**, *184*, 83–85.
